# Genetic deletion of MrgD receptor disrupts cardiac protein homeostasis in mice

**DOI:** 10.1007/s11033-026-11639-8

**Published:** 2026-03-07

**Authors:** Beatriz Alexandre-Santos, Luiza Mazzali Ferraz, Ana Beatriz Proença, Nícia Pedreira Soares, Guilherme dos Santos Reis, Maria Eduarda Lima da Silva, D’Angelo Carlo Magliano, Maria Jose Campagnole-Santos, Antonio Claudio Lucas da Nóbrega, Robson Augusto Souza Santos, Eliete Dalla Corte Frantz

**Affiliations:** 1 Laboratory of Exercise Sciences, Biomedical Institute, Fluminense Federal University, Niteroi, RJ Brazil; 2 Research Center on Morphology and Metabolism, Biomedical Institute, Fluminense Federal University, Niteroi, RJ Brazil; 3 National Institute of Science and Technology - INCT Physical (In)activity and Health, CNPq, Brazil; 4 Department of Physiology and Biophysics, Institute of Biological Sciences, National Institute of Science and Technology in Nanobiopharmaceutics, Federal University of Minas Gerais, Belo Horizonte, Brazil; 5 Department of Morphology, Biomedical Institute, Fluminense Federal University, Rua Alameda Barros Terra, s/n, Sao Domingos, Niteroi, 24.020-150 RJ Brazil

**Keywords:** Renin-angiotensin system (RAS), Mas-related G-protein coupled receptor type D (MrgD), Cardiac remodeling, Protein homeostasis, Protein degradation

## Abstract

**Background:**

Cardiovascular diseases are the leading cause of death worldwide. An important mechanism involved is the disruption in protein homeostasis by overactivation of the classical axis of the renin-angiotensin system. The counterregulatory axis counteracts these effects; however, the MrgD receptor has recently been described, and its effects are unknown. Thus, this study aims to evaluate the impact of MrgD deficiency on cardiac protein homeostasis.

**Methods:**

16-week-old wild-type (WT) and MrgD knockout (MrgD KO) male C57BL/6J mice were evaluated for systolic blood pressure (SBP), cardiac morphology, MDA levels, carbonyl content, and protein homeostasis markers.

**Results:**

SBP and heart mass remained unaltered. MrgD deficiency increased left ventricular mass and led to cardiac atrophy by reduced left ventricular wall thickness and cardiomyocyte cross-sectional area. Collagen (types 1 and 3) deposition and MMP-2 cardiac protein expression were elevated in MrgD KO. Genetic deletion of MrgD increased NOX2, NOX4, and ERO1α cardiac protein expression. MDA levels were similar between groups, and carbonyl content was higher in MrgD KO. GRP78, CHOP, MuRF-1, Atrogin-1, and polyubiquitinated proteins were increased in MrgD KO mice, indicating a loss of protein homeostasis.

**Conclusions:**

The genetic deletion of MrgD promoted cardiac remodeling and disrupted protein homeostasis, with increased pro-oxidative response and ER stress. This was associated with protein degradation by the activation of the ubiquitin-proteasome pathway.

**Supplementary Information:**

The online version contains supplementary material available at 10.1007/s11033-026-11639-8.

## Introduction

Cardiovascular diseases (CVD) are the leading cause of death worldwide, accounting for an increasing number of deaths each year [1]. This alarming epidemiological scenario brings attention to CVD as a major global public health burden. The development and progression of CVD are multifactorial, involving a complex interplay of several pathophysiological mechanisms [2]. Overactivation of the classical axis of the renin-angiotensin system (RAS) plays a central role in the pathogenesis of several cardiovascular disorders, including hypertension, heart failure, and atherosclerosis [3, 4].

The classical axis of the RAS is composed of Angiotensin Converting Enzyme (ACE), Angiotensin (Ang)-II, and the Ang-II type 1 receptor (AT1R) [5]. When overactivated, this axis induces cardiac remodeling, encompassing hypertrophy and fibrosis, through the disruption of several cellular processes [3, 6]. These processes include excessive generation of reactive oxygen species (ROS), leading to a pro-oxidative environment that overloads cardiomyocyte protein homeostasis. Consequently, protein synthesis, folding, and quality control mechanisms within the endoplasmic reticulum (ER) are impaired [6–8]. Sustained ER stress further exacerbates proteostatic imbalance, favoring protein degradation by regulating proteolytic pathways within the ubiquitin-proteasome system [8, 9].

On the other hand, the counterregulatory axis of the RAS is traditionally composed of ACE2, Ang- [1–7], and Mas receptor (MasR) [5]. This pathway counteracts the deleterious effects of the classical axis by reducing ROS formation and attenuating disturbances in cardiac protein homeostasis [10, 11]. More recently, in 2013, a novel counterregulatory RAS pathway was identified: the alamandine/Mas-related G-protein coupled receptor type D (MrgD) pathway [12]. This pathway appears to be involved in maintaining cardiac shape and function [13, 14]. Alamandine treatment has been suggested to exert anti-inflammatory and antifibrotic effects [15], but the actions of this peptide might be mediated by other receptors or heterodimerization [14, 16]. Experimental data have demonstrated that the genetic deletion of MrgD induces dilated cardiomyopathy, underscoring its importance for cardiac integrity [17]. However, the molecular mechanisms underlying the potential cardioprotective actions of MrgD remain unexplored. Thus, this study aims to evaluate whether MrgD deficiency is involved in regulating cardiac pro-oxidative responses and protein homeostasis.

## Methods

### Experimental protocol

Animal care and procedures followed guidelines established in the National Institutes of Health Guide (8th edition, 2011). All experiments were approved by the Animal Ethics Committee of the Federal University of Minas Gerais (protocol number 2132023). The experimental protocol was carried out at the Transgenic Animal Facility, Hypertension Laboratory, Federal University of Minas Gerais, Brazil. Wild-type C57BL/6J (WT) and MrgD *knockout* (MrgD KO) mice (n = 10/group) were purchased from the Mutant Mouse Regional Resource Center (National Institutes of Health, RRID: MMRRC_036050-UNC). MrgD homozygous deficiency was validated using the following primers: MrgD-8, 5’-CATGAGATGCTCTATCCATTGGG-3’; reverse tetracycline transactivator primer (rtTA1), 5’-GGAGAAACAGTCAAAGTGCG-3’; and MrgD-1, 5’-CTGCTCATAGTCAACATTTCTGC-3’. Animals were housed in a temperature-controlled room (23 ± 2 °C) with a 12:12-h light-dark cycle, provided with food and water *ad libitum*.

Systolic blood pressure (SBP) was measured at 8 and 16 weeks of age by tail-cuff plethysmography (CODA, *Kent Scientific*, USA). At least 1 week before SBP recording, mice were acclimated for three consecutive days.

### Tissue extraction

At 16 weeks old, animals were fasted for six hours, heparinized, deeply anesthetized with intraperitoneal ketamine (40 mg/kg) and xylazine (8 mg/kg), and euthanized by exsanguination. Hearts were weighed, and left ventricles (LV) were dissected, weighed, and then embedded in buffered formalin for histological analysis (*n* = 5 animals/group), or rapidly frozen and stored at −80°C until subsequent analysis (*n* = 5 animals/group).

### Histological analysis

Fixed LV samples were embedded in paraffin, and 5-µm-thick sections were stained with either hematoxylin and eosin or Picrosirius Red. Digital images were obtained using the ScanScopeTM CS (Aperio Technologies, Leica, CA, USA).

In hematoxylin and eosin-stained slides, LV wall thickness and LV chamber area were assessed. Fifteen digital images, at least 10 cardiomyocytes per image, were analyzed per animal to estimate cardiomyocyte area using the digital image analysis system Image Pro Plus (version 4.5, Media Cybernetics, MD, USA). Cardiomyocyte cross-sectional area was estimated based on the numerical density of cardiomyocytes, in a frame of known area, produced by STEPanizer Web-based software [18].

In Picrosirius Red-stained slides, fifteen digital images were analyzed per animal to estimate collagen deposition. These measurements were evaluated using the digital image analysis system Image Pro Plus (version 4.5, Media Cybernetics, MD, USA) [19].

### Immunohistochemistry

Fixed LV samples (*n* = 5/group) were embedded in paraffin, and 5 μm-thick sections were submitted to antigen retrieval and blockade (Novolink Polymer Detection Systems Kit; Leica Biosystems, RU), as described previously [20]. LV sections were incubated overnight at 4°C with primary antibodies for collagen type 1 (PAE-95137, Invitrogen) and collagen type 3 (PAD195Mu02, Cloud Clone Corp), followed by incubation with Envision FLEX/HRP (DAKO, 152 Agilent, SM802) for signal amplification. Diaminobenzidine was used for the detection of positive immunoreaction, and hematoxylin was used for counterstaining. Negative controls were performed by omitting the primary antibody. Digital images were obtained using the ScanScope CS (Aperio Technologies, CA, USA). The immunoreactivity areas were estimated using the Image-Pro Plus (Media Cybernetics, Silver Spring, MD, USA), through the density threshold selection tool. The results were presented as a percentage of the WT group [21, 22].

### Western blot

Total LV proteins were extracted (*n* = 5/group), and protein samples were resolved by SDS-PAGE 10–15% gel electrophoresis and transferred to a polyvinylidene difluoride membrane. Membranes were incubated overnight at 4°C with primary antibodies for: matrix metalloproteinase 2 (MMP-2, PAA100Hu01, Cloud-Clone Corp), NADPH oxidase (NOX) 2 (sc-5826, Santa Cruz Biotechnology), NOX4 (sc-30141, Santa Cruz Biotechnology), endoplasmic oxidoreductin-1α (ERO1α, sc-365526, Santa Cruz Biotechnology), C/EBP-homologous protein (CHOP, sc-71136, Santa Cruz Biotechnology), 78 kDa glucose-regulated protein (GRP78, CSB-PA873500, Cusabio Biotech Co), Muscle Ring Finger protein-1 (MuRF-1, FNab08992, FineTest Biotech Inc), Atrogin-1 (PAF435Hu01, Cloud-Cone Corp), Ubiquitin (sc-166553, Santa Cruz Biotechnology). Secondary antibodies and ECL Western blot reagents were used to detect the binding of the primary antibodies. Images were acquired using the ChemiDoc system (Bio-Rad, Hercules, CA, USA). The intensity of the chemiluminescent bands was quantified using ImageJ software, version 1.44 (NIH, imagej.nih.gov/ij, USA). β-actin was used as a loading control.

### Real-time reverse transcriptase polymerase chain reaction (RT-qPCR)

Total RNA was extracted from 40 mg of LV using a Trizol reagent (Invitrogen, CA, USA). RNA concentration and integrity were assessed, and synthesis of first-strand cDNA was performed. RNA samples were quantified by absorbance at 260 nm and 280 nm to determine concentration and purity. Quantitative real-time PCR was performed using an Applied Biosystems thermocycler (7500 Fast, Thermo Fisher Scientific, CA, USA) and GoTaq^®^ qPCR Master Mix (Promega, MA, USA). Constitutive gene expression (TATA box-binding protein, Tbp) was coamplified with the samples and quantified as an endogenous control. All samples were assayed in duplicate. The results were quantified by the 2 -ΔΔCt method. The primers used in this study are listed in Table S1.

### Thiobarbituric acid reactive substances (TBARS) assay

Lipid peroxidation in LV homogenate (*n* = 5/group) was evaluated by measuring the level of thiobarbituric acid–reactive substances (TBARS) following the method of Ohkawa et al. [23]. Malondialdehyde (MDA) concentration was determined from absorbance at 532 nm and presented as MDA/mg of protein.

### Protein carbonylation

Oxidative damage to proteins was measured based on the reaction between LV homogenate and dinitrophenylhydrazine (*n* = 5/group), as previously described [24]. Carbonyl content was determined from absorbance at 370 nm and expressed as carbonyl/mg of protein.

### Statistical analysis

Data are presented as means ± standard deviation (SD). Data were tested for normality and homoscedasticity using the Shapiro-Wilk test. The differences between groups were determined by an unpaired Student’s t-test with Welch’s correction. In all cases, *p* < 0.05 was considered statistically significant. Analysis was performed by GraphPad Prism (version 8.0.2, La Jolla, CA, USA).

## Results

### MrgD deficiency induces cardiac atrophy

**Fig. 1:**
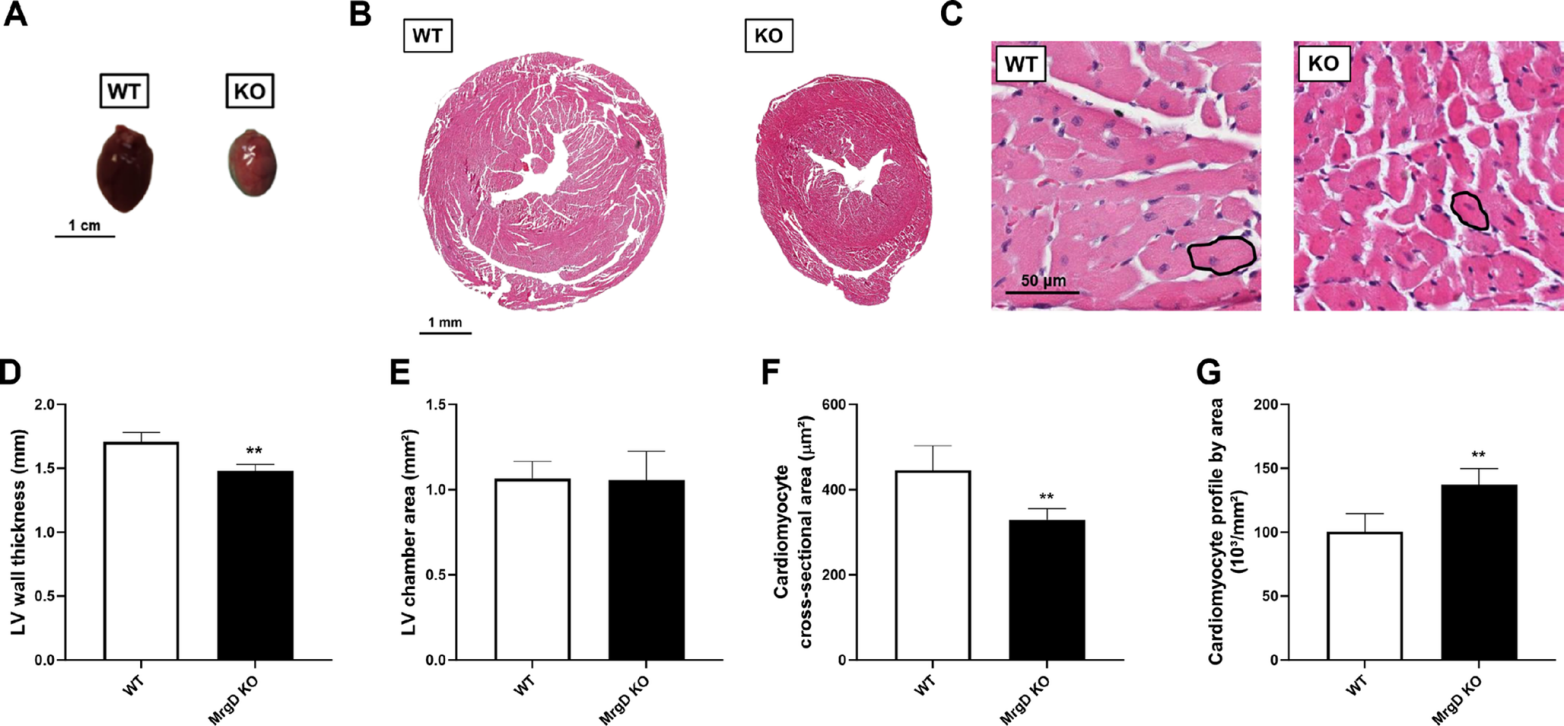
Evaluation of cardiac morphology. (**A**) Macroscopic view of the whole heart (bar = 1 cm). (**B**, **C**) Representative photomicrographs of hematoxylin and eosin-stained histological sections of the heart in low (**B**, bar = 1 mm) and high (**C**, bar = 50 μm, with cross-sectional cardiomyocytes) magnifications. (**D**) LV wall thickness. (**E**) LV chamber area. (**F**) Cardiomyocyte cross-sectional area. (**G**) Cardiomyocyte profile by area. Data presented as mean ± SD (n = 5/group). Significant differences between groups are indicated with the symbols: ** ≠ WT (p < 0.01), as determined by unpaired Student’s t test with Welch’s correction

SBP was similar between groups at both 8 and 16 weeks (Table 1). Genetic deletion of MrgD did not affect absolute and relative heart mass but increased absolute (+ 12.35%, *p* < 0.05) and relative (+ 12.72%, *p* < 0.05) LV mass (Table 1). Cardiac size was smaller in the MrgD KO group in qualitative macroscopy (Fig. 1A). LV wall was 13.27% thinner in the MrgD KO group (*p* < 0.01) (Fig. 1B and D). The LV chamber area remained similar between groups (Fig. 1B and E). Cardiomyocyte cross-sectional area was smaller in the MrgD KO group (−26.14%, *p* < 0.01; Fig. 1C and F). Consistently, MrgD deficiency increased cardiomyocyte profile by area (+ 36.54%, *p* < 0.01; Fig. 1C and G).

**Table 1:** Biometric parameters

	WT	MrgD KO
SBP (mmHg), week 8	123.7 ± 10.73	127.2 ± 9.83
SBP (mmHg), week 16	122.3 ± 14.76	122.2 ± 14.53
Heart Mass (mg)	134.1 ± 17.13	140.4 ± 17.75
Heart Mass/tibia (mg/cm)	76,0 ± 10,87	79,2 ± 10,11
LV Mass (mg)	92.3 ± 12.52	103.7 ± 8.63^*^
LV Mass/tibia (mg/cm)	51,9 ± 4,89	58,5 ± 5,12^*^

Data presented as mean ± standard deviation (*n* = 10/group). Significant differences between groups are indicated with the symbols: * ≠ WT (*p* < 0.05), as determined by unpaired Student’s t test with Welch’s correction

### Genetic deletion of MrgD increases cardiac collagen deposition

**Fig. 2:**
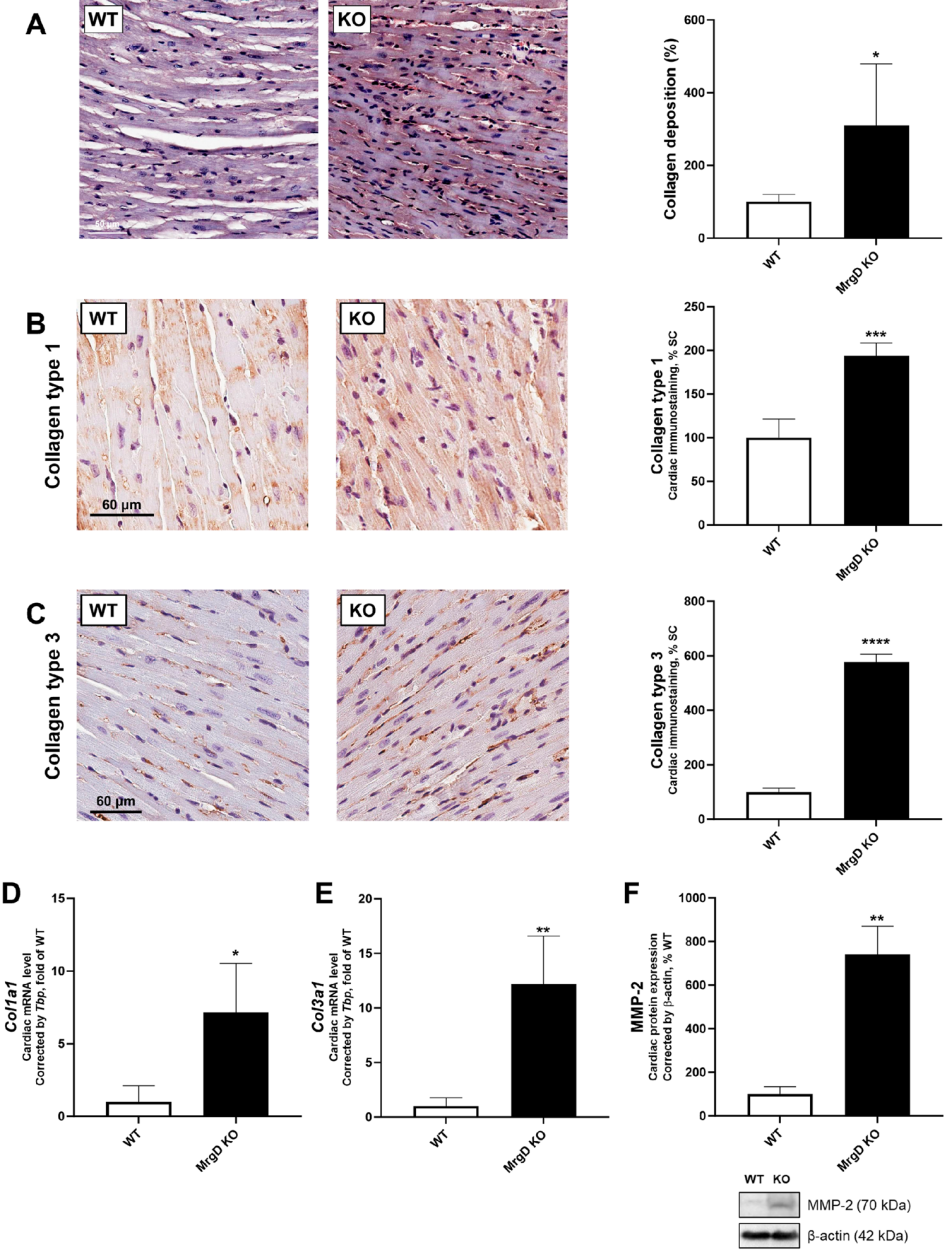
Evaluation of cardiac collagen deposition. (**A**) Representative photomicrographs of Picrosirius Red-stained histological sections of the heart in the same magnification (bar = 50 μm), and collagen deposition. (**B**) Representative photomicrographs of collagen type 1 immunostaining counterstained with hematoxylin in cardiac tissue at the same magnification in all groups (bar = 60 μm). (**C**) Representative photomicrographs of collagen type 3 immunostaining counterstained with hematoxylin in cardiac tissue at the same magnification in all groups (bar = 60 μm). (**D**) *Col1a1* and (**E**) *Col3a1* mRNA expression. (**F**) MMP-2 protein expression and representative Western blot analysis of proteins in LV. Data presented as mean ± SD (*n* = 5/group). Significant differences between groups are indicated with the symbols: * ≠ WT (*p* < 0.05), *** ≠ WT (*p* < 0.001), **** ≠ WT (*p* < 0.0001), as determined by unpaired Student’s t test with Welch’s correction

In Picrosirius-stained slides, the MrgD KO group exhibited 209.28% higher collagen deposition (*p* < 0.05) (Fig. 2A). Accordingly, genetic deletion of MrgD increased collagen type 1 immunostaining (+ 93.30%, *p* < 0.001) and mRNA expression (+ 614.26%, *p* < 0.05) (Fig. 2B and D). Collagen type 3 immunostaining (+ 476.25%, *p* < 0.0001) and mRNA expression (+ 1121.50%, *p* < 0.01) were also augmented in the MrgD KO group (Fig. 2C and E). Cardiac MMP-2 protein expression was augmented in the MrgD KO group (+ 641.06%, *p* < 0.01; Fig. 2F).

### A pro-oxidative environment is related to MrgD deficiency

**Fig. 3:**
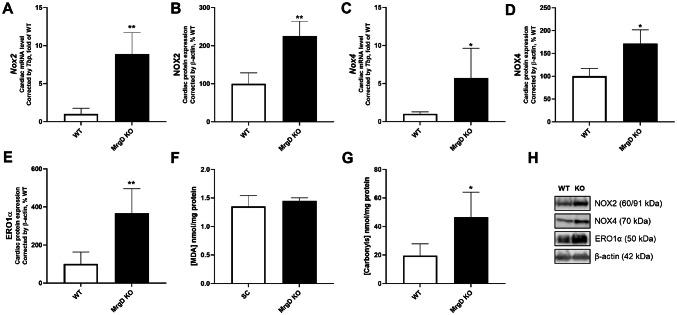
Evaluation of pro-oxidative response. NOX2 (**A**) mRNA and (**B**) protein expression. NOX4 (**C**) mRNA and (**D**) protein expression. (**E**) ERO1α protein expression. (**F**) MDA levels. (**G**) Protein carbonyl content. (**H**) Representative Western blot analysis of proteins in LV. Data presented as mean ± SD (*n* = 5/group). Significant differences between groups are indicated with the symbols: * ≠ WT (*p* < 0.05), ** ≠ WT (*p* < 0.01), as determined by unpaired Student’s t test with Welch’s correction

Genetic deletion of MrgD increased cardiac mRNA (+ 786.26%, *p* < 0.01) and protein expression (+ 125.04%, *p* < 0.01) of NOX2 (Fig. 3A, B and H). NOX4 mRNA (+ 470.70%, *p* < 0.05) and protein expression (+ 71.75%, *p* < 0.05) were also increased in the MrgD KO group (Fig. 3C, D and H). In addition, cardiac ERO1α protein expression was elevated in the MrgD KO group (+ 265.73%, *p* < 0.01) (Fig. 3E and H). Although MDA levels did not differ between groups, the MrgD KO group had higher carbonyl content (+ 139.15%, *p* < 0.05; Fig. 3F and G).

### MrgD deficiency increases ER stress and ubiquitin-related protein degradation

**Fig. 4:**
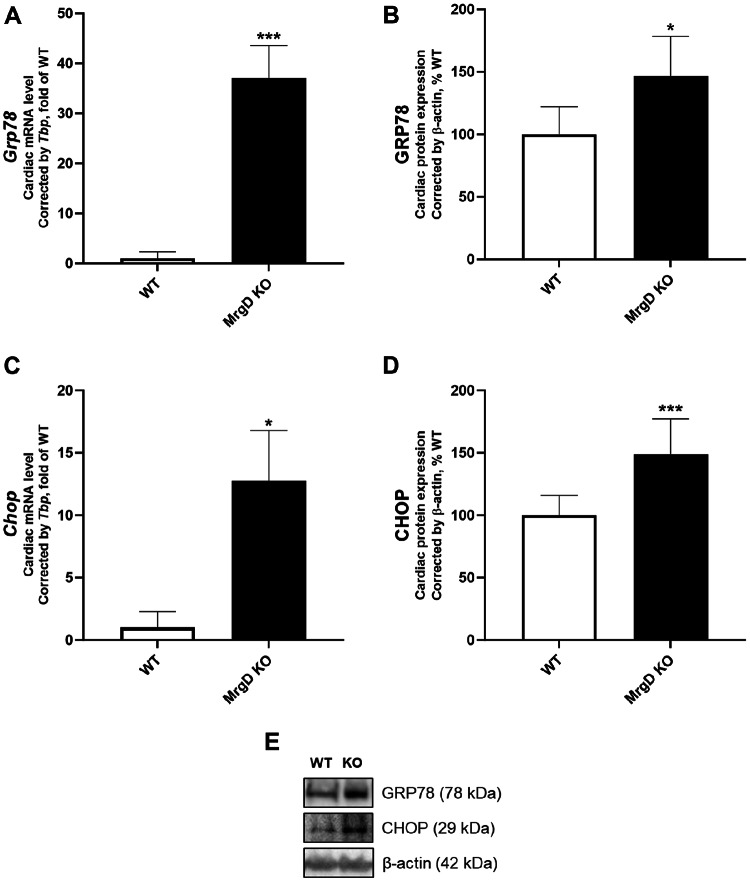
Evaluation of ER stress. GRP78 (**A**) mRNA and (**B**) protein expression. CHOP (**C**) mRNA and (**D**) protein expression. (**E**) Representative Western blot analysis of proteins in LV. Data presented as mean ± SD (*n* = 5/group). Significant differences between groups are indicated with the symbols: * ≠ WT (*p* < 0.05), ** ≠ WT (*p* < 0.01), as determined by unpaired Student’s t test with Welch’s correction

Regarding ER stress, cardiac GRP78 mRNA (+ 3605.90%, *p* < 0.001) and protein (+ 46.27%, *p* < 0.05) expression were higher in the MrgD KO group (Fig. 4A, B and E). Cardiac CHOP mRNA (+ 1174.30%, *p* < 0.001) and protein expression (+ 49.03%, *p* < 0.05) were also augmented in the MrgD KO group (Fig. 4C, D and E).

**Fig. 5:**
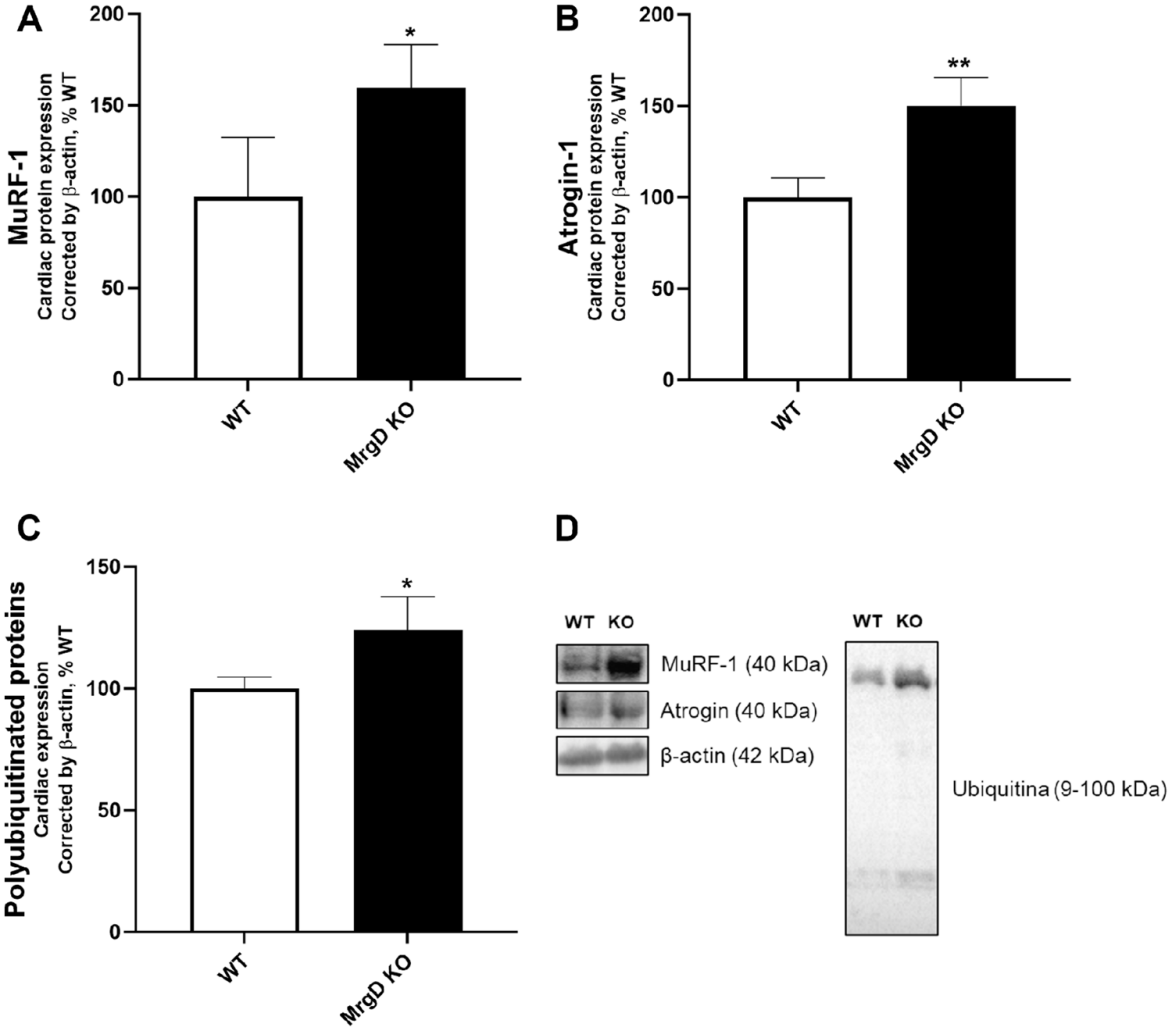
Evaluation of protein homeostasis. (**A**) MuRF-1, (**B**) Atrogin-1, and (**C**) Polyubiquitinated proteins expression. (**D**) Representative Western blot analysis of proteins in LV. Data presented as mean ± SD (*n* = 5/group). Significant differences between groups are indicated with the symbols: * ≠ WT (*p* < 0.05), ** ≠ WT (*p* < 0.01), as determined by unpaired Student’s t test with Welch’s correction

Consistent with these findings, MrgD deficiency increased cardiac protein expression of MuRF-1 (+ 59.54%, *p* < 0.05) and Atrogin-1 (+ 50.18%, *p* < 0.01), muscle-specific E3 ligases of the ubiquitin-proteasome pathway (Fig. 5A, B and D). Moreover, the MrgD KO group had higher polyubiquitinated proteins (+ 23.94%, *p* < 0.05; Fig. 5C and D).

## Discussion

**Fig. 6:**
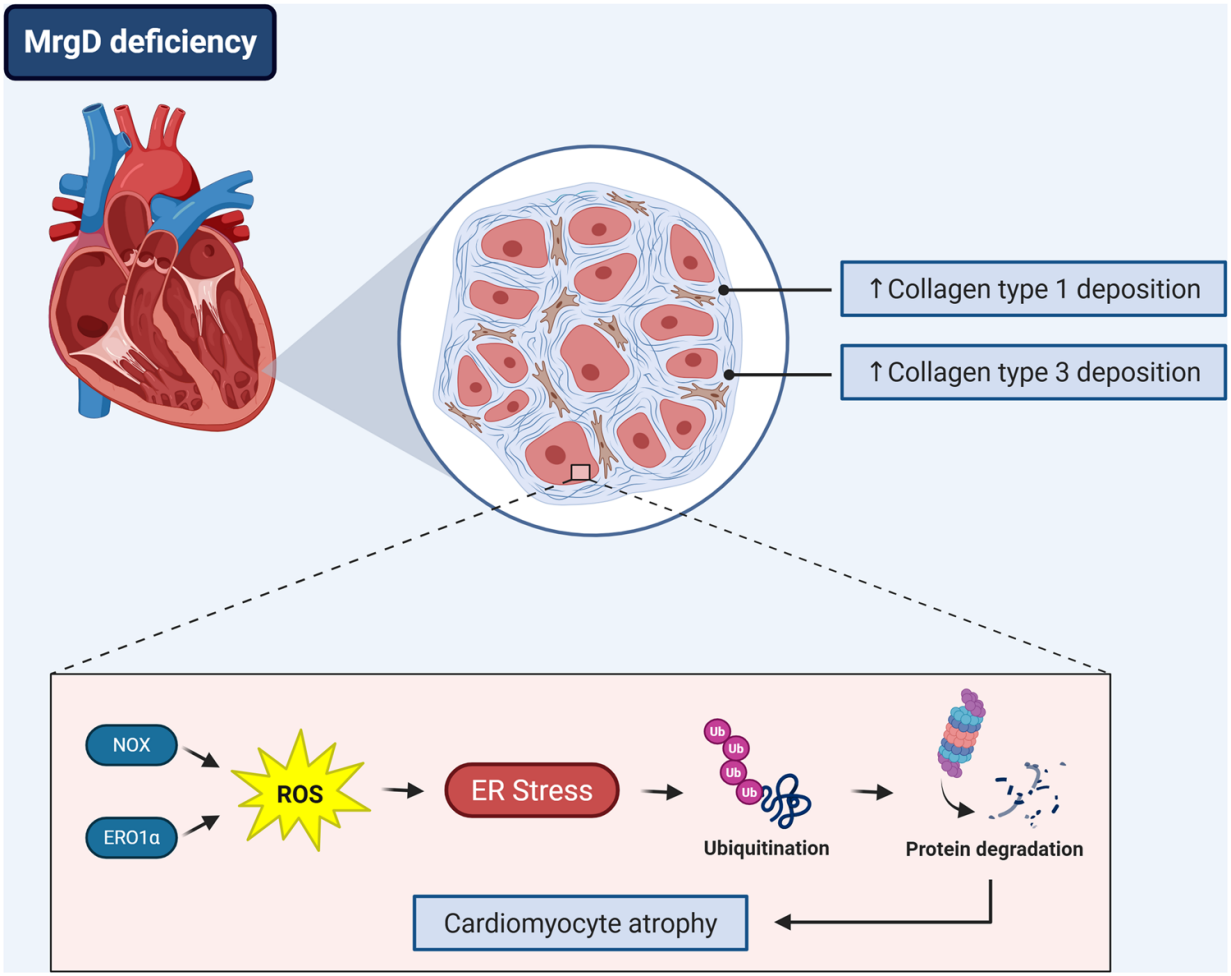
MrgD deficiency disrupts cardiac protein homeostasis. MrgD deficiency led to a pattern of dilated cardiomyopathy with a remodeling process characterized by cardiac atrophy and fibrosis. Changes in cardiac extracellular matrix were evidenced by increased collagen types 1 and 3 deposition. The atrophic response was associated with increased protein degradation, which occurred as a result of a disruption in protein homeostasis. MrgD deletion increased ROS by augmenting both NOX and ERO1α protein expression, which increased ER stress and, consequently, the ubiquitin-proteasome pathway

Genetic deletion of the MrgD receptor induced cardiac atrophy and collagen deposition, possibly due to a loss of protein homeostasis, as evidenced by the promotion of a pro-oxidative environment and ER stress. This led to the activation of the ubiquitin-proteasome pathway, a major mechanism of protein degradation (Fig. 6).

Regarding SBP, we did not find any difference between groups. This contrasts with the previously described anti-hypertensive effects of alamandine through MrgD [12, 25, 26]. However, this is only the second study to evaluate SBP in MrgD KO animals. Oliveira et al. reported similar SBP between 12-week-old WT and MrgD KO animals, supporting our findings [17].

Consistent with the atrophic response observed herein, a previous study demonstrated, using echocardiographic evaluation, that MrgD deficiency led to dilated cardiomyopathy [17]. Although we did not observe LV chamber dilation, possibly due to a lack of hemodynamic factors, such as intracardiac pressure, in histological analysis, our findings corroborate this LV remodeling. Despite the atrophy, heart mass was not altered, and LV mass was augmented. This discrepancy between size and weight might be explained by organ histoarchitecture. Although smaller, the hearts of MrgD KO animals had increased cardiac collagen expression and deposition, and collagen is denser than normal interstitium. This is the first study to directly assess heart weight and extracellular matrix features in MrgD KO animals. However, Oliveira et al. found, by echocardiography, that genetic deletion of MrgD did not alter ventricular mass in 2-week-old mice but increased this parameter in 12-week-old mice [17]. The pro-fibrotic effect of MrgD deficiency is consistent with the reported anti-fibrotic effect of alamandine. In different models of myocardial infarction and hypertension, alamandine treatment has been shown to reduce collagen deposition and MMP-2 and transforming growth factor-β (TGF-β) expression [27–29]. Although our findings suggest a pattern of structural remodeling that is commonly associated with cardiac dysfunction and increased myocardial stiffness, the lack of functional analysis limits the confirmation of dilated cardiomyopathy as previously described [17]. Despite that, similar morphological responses were observed, corroborating the intimate relation played by function and morphology in cardiac pathophysiology [30].

This pronounced LV remodeling upon MrgD deficiency was accompanied by a pro-oxidative environment, with increased ROS formation resulting from both superoxide production by NOX and hydrogen peroxide by ERO1α. Oxidative damage may result from lipid peroxidation, protein oxidation, and DNA oxidation [31]. Genetic deletion of MrgD augmented protein oxidation, but lipid peroxidation remained unaltered. Interestingly, a pro-oxidative response is known to contribute to the sustained accumulation of unfolded and misfolded proteins, thus triggering the unfolded protein response (UPR) and ER stress [8]. Indeed, ER stress markers were increased in MrgD KO animals. This is the first study to report any impact of MrgD on ER stress, and studies addressing other counterregulatory components remain scarce. In agreement with our findings, few data available suggest that reducing the Ang- [1–7]/MasR pathway leads to UPR activation [8], indicating an involvement of the counterregulatory axis in ER stress regulation.

The UPR is a key regulator of proteolytic pathways, particularly the ubiquitin-proteasome system, aiming to reestablish protein homeostasis [32]. In the heart, this process depends on muscle-specific E3 ligases, i.e., MuRF-1 and Atrogin-1, which are hallmarks of muscle atrophy [33–35]. These markers were increased in MrgD KO animals, which suggests activation of the ubiquitin-proteasome pathway. Corroborating these findings, MrgD deficiency increased polyubiquitinated proteins. The role of the counterregulatory axis in muscle atrophy is poorly explored. In skeletal muscle, the Ang- [1–7]/MasR pathway appears to have a protective role, reducing MuRF-1 and Atrogin-1, and preventing protein polyubiquitination [36, 37]. To date, this is the first study to associate MrgD with markers of protein degradation.

Although this study only evaluated male mice, the previous study characterizing dilated cardiomyopathy in MrgD-deficient mice observed that this effect is independent of sex [17]. Thus, this does not blunt the relevance of the findings described herein.

## Conclusions

Taken together, our findings demonstrate that the cardiac effects of genetic deletion of MrgD are associated with disturbances in protein homeostasis, characterized by an increase in protein degradation. This may contribute to the structural alterations observed, with cardiac atrophy and fibrosis. Importantly, these results further reinforce the protective role of MrgD in maintaining cardiac integrity, suggesting that its presence is essential for preserving protein stability.

## Supplementary Information

Below is the link to the electronic supplementary material.

Supplementary Material 1

## Data Availability

Data is available on request from the authors.The data supporting this study’s findings are available from the corresponding author upon reasonable request. Some data may not be made available because of privacy or ethical restrictions.

## Abbreviations

Ang: Angiotensin
ACE: Angiotensin converting enzyme
AT1R: Ang-II type 1 receptor
CVD: Cardiovascular diseases
CHOP: C/EBP-homologous protein
Col1a1: Collagen type 1 alpha 1 chain
Col3a1: Collagen type 3 alpha 1 chain
ER: Endoplasmic reticulum
ERO1α: Endoplasmic oxidoreductin-1α
LV: Left ventricle
MDA: Malondialdehyde
MasR: Mas receptor
MrgD: Mas-related G-protein coupled receptor type D
MMP-2: Matrix metalloproteinase 2
MrgD KO: MrgD knockout
MuRF-1: Muscle ring finger protein-1
NOX: NADPH oxidase
ROS: Reactive oxygen species
RAS: Renin-angiotensin system
RT-qPCR: Real-time reverse transcriptase polymerase chain reaction
SD: Standard deviation
SBP: Systolic blood pressure
TBARS: Thiobarbituric acid reactive substances
TGF-β: Transforming growth factor-β
UPR: Unfolded protein response
WT: Wild type
GRP78: 78 kDa glucose-regulated protein

## References

[1] Di Cesare M, Perel P, Taylor S, Kabudula C, Bixby H, Gaziano TA, McGhie DV, Mwangi J, Pervan B, Narula J, Pineiro D, Pinto FJ (2024) The heart of the world. Glob Heart 19(1):11. https://pmc.ncbi.nlm.nih.gov/articles/PMC10809869/38273998 10.5334/gh.1288 PMC10809869

[2] Schirone L, Forte M, Palmerio S, Yee D, Nocella C, Angelini F, Pagano F, Schiavon S, Bordin A, Carrizzo A, Vecchione C, Valenti V, Chimenti I, De Falco E, Sciarretta S, Frati G (2017) A Review of the Molecular Mechanisms Underlying the Development and Progression of Cardiac Remodeling. Oxid Med Cell Longev 2017:3920195. 10.1155/2017/3920195 28751931 10.1155/2017/3920195 PMC5511646

[3] Ames MK, Atkins CE, Pitt B (2019) The renin-angiotensin-aldosterone system and its suppression. J Vet Intern Med 33(2):363–382. https://onlinelibrary.wiley.com/doi/full/10.1111/jvim.15454 30806496 10.1111/jvim.15454 PMC6430926

[4] Schmieder RE, Hilgers KF, Schlaich MP, Schmidt BM (2007) Renin-angiotensin system and cardiovascular risk. Lancet 369(9568):1208–1219. https://pubmed.ncbi.nlm.nih.gov/17416265/17416265 10.1016/S0140-6736(07)60242-6

[5] Bader M, Muscha Steckelings U, Alenina N, Santos RAS, Ferrario CM (2024) Alternative Renin-Angiotensin System. Hypertens (Dallas, Tex 1979) https://pubmed.ncbi.nlm.nih.gov/38362781/10.1161/HYPERTENSIONAHA.123.21364 PMC11023806 38362781

[6] Azevedo PS, Polegato BF, Minicucci MF, Paiva SAR, Zornoff LAM (2016) Cardiac Remodeling: Concepts, Clinical Impact, Pathophysiological Mechanisms and Pharmacologic Treatment. Arquivos brasileiros de cardiologia 106:62–69 10.5935/abc.20160005 PMC4728597 26647721

[7] Kny M, Fielitz J (2022) Hidden Agenda - The Involvement of Endoplasmic Reticulum Stress and Unfolded Protein Response in Inflammation-Induced Muscle Wasting. Front Immunol 13:878755. 10.3389/fimmu.2022.878755 35615361 10.3389/fimmu.2022.878755 PMC9124858

[8] Sepúlveda-Fragoso V, Alexandre-Santos B, Salles ACP, Proença AB, de Paula Alves AP, Vázquez-Carrera M, Nóbrega ACL, Frantz EDC, Magliano DC (2021) Crosstalk between the renin-angiotensin system and the endoplasmic reticulum stress in the cardiovascular system: Lessons learned so far. Life Sci 284:119919. https://linkinghub.elsevier.com/retrieve/pii/S0024320521009061 34480931 10.1016/j.lfs.2021.119919

[9] Ji Y, Jiang Q, Chen B, Chen X, Li A, Shen D, Shen Y, Liu H, Qian X, Yao X, Sun H (2025) Endoplasmic reticulum stress and unfolded protein response: Roles in skeletal muscle atrophy. Biochem Pharmacol 234:116799. https://pubmed.ncbi.nlm.nih.gov/39952329/39952329 10.1016/j.bcp.2025.116799

[10] Murugan D, Lau YS, Lau WC, Mustafa MR, Huang Y (2015) Angiotensin 1–7 protects against angiotensin II-induced endoplasmic reticulum stress and endothelial dysfunction via mas receptor. PLoS One. 10.1371/journal.pone.0145413 26709511 10.1371/journal.pone.0145413 PMC4692500

[11] Santos RAS, Sampaio WO, Alzamora AC, Motta-Santos D, Alenina N, Bader M, Campagnole-Santos MJ (2018) The ACE2/Angiotensin-(1-7)/MAS Axis of the Renin-Angiotensin System: Focus on Angiotensin-(1-7). Physiol Rev 98(1):505–553. https://pubmed.ncbi.nlm.nih.gov/29351514/29351514 10.1152/physrev.00023.2016 PMC7203574

[12] Lautner RQ, Villela DC, Fraga-Silva RA, Silva N, Verano-Braga T, Costa-Fraga F et al (2013) Discovery and characterization of alamandine: A novel component of the renin-angiotensin system. Circ Res 112(8):1104–1111 23446738 10.1161/CIRCRESAHA.113.301077

[13] Gamiño-Gutiérrez JA, Terán-Hernández IM, Castellar-Lopez J, Villamizar-Villamizar W, Osorio-Llanes E, Palacios-Cruz M, Rosales W, Chang AY, Díaz-Ariza LA, Ospino MC, Mendoza-Torres E (2024) Novel Insights into the Cardioprotective Effects of the Peptides of the Counter-Regulatory Renin-Angiotensin System. Biomedicines 12(2):255. https://pubmed.ncbi.nlm.nih.gov/38397857/38397857 10.3390/biomedicines12020255 PMC10887066

[14] Jesus ICG, Mesquita T, Souza Santos RA, Guatimosim S (2023) An overview of alamadine/MrgD signaling and its role in cardiomyocytes. Am J Physiol Cell Physiol 324(3):C606-C613. https://pubmed.ncbi.nlm.nih.gov/36571443/10.1152/ajpcell.00399.2021 PMC11033694 36571443

[15] Ávila-Martínez DV, Mixtega-Ruiz WK, Hurtado-Capetillo JM, Lopez-Franco O, Flores-Muñoz M (2024) Counter-regulatory RAS peptides: new therapy targets for inflammation and fibrotic diseases? Front Pharmacol 15:1377113 38666016 10.3389/fphar.2024.1377113 PMC11044688

[16] Rukavina Mikusic NL, Silva MG, Erra Díaz FA, Pineda AM, Ferragut F, Gómez KA, Mazzitelli L, Gonzalez Maglio DH, Nuñez M, Santos RAS, Grecco HE, Gironacci MM (2024) Alamandine, a protective component of the renin-angiotensin system, reduces cellular proliferation and interleukin-6 secretion in human macrophages through MasR-MrgDR heteromerization. Biochem Pharmacol 229:116480. https://pubmed.ncbi.nlm.nih.gov/39128587/39128587 10.1016/j.bcp.2024.116480

[17] Oliveira AC, Melo MB, Motta-Santos D, Peluso AA, Souza-Neto F, Da Silva RF et al Genetic deletion of the alamandine receptor MRGD leads to dilated cardiomyopathy in mice. Am J Physiol Heart Circ Physiol [Internet]. 2019 Jan 1 [cited 2024 Mar 27];316(1):H123–33. Available from: https://pubmed.ncbi.nlm.nih.gov/30339496/10.1152/ajpheart.00075.2018 30339496

[18] Barbosa-da-Silva S, Fraulob-Aquino JC, Lopes JR, Mandarim-de-Lacerda CA, Aguila MB (2012) Weight cycling enhances adipose tissue inflammatory responses in male mice. PLoS One [Internet]. Jul 25 [cited 2025 Jul 3];7(7). Available from: https://pubmed.ncbi.nlm.nih.gov/22848362/10.1371/journal.pone.0039837 PMC3405086 22848362

[19] Mandarim-de-Lacerda CA, Fernandes-Santos C, Aguila MB (2010) Image analysis and quantitative morphology. Methods Mol Biol 611:211–225. https://pubmed.ncbi.nlm.nih.gov/19960334/19960334 10.1007/978-1-60327-345-9_17

[20] Alexandre-Santos B, Machado MV, Menezes AC, Velasco LL, Sepúlveda-Fragoso V, Vieira AB et al (2019) Exercise-induced cardiac opioid system activation attenuates apoptosis pathway in obese rats. Life Sci. 10.1016/j.lfs.2019.06.017 31176781 10.1016/j.lfs.2019.06.017

[21] Mandarim-de-Lacerda CA, Fernandes-Santos C, Aguila MB (2010) Image analysis and quantitative morphology. Methods Mol Biol 611:211-225. https://link.springer.com/protocol/10.1007/978-1-60327-345-9_17 10.1007/978-1-60327-345-9_17 19960334

[22] Frantz ED, Crespo-Mascarenhas C, Barreto-Vianna AR, Aguila MB, Mandarim-de-Lacerda CA (2013) Renin-angiotensin system blockers protect pancreatic islets against diet-induced obesity and insulin resistance in mice. PLoS ONE 8(7):e67192. https://pubmed.ncbi.nlm.nih.gov/23894285/23894285 10.1371/journal.pone.0067192 PMC3718820

[23] Ohkawa H, Ohishi N, Yagi K (1979) Assay for lipid peroxides in animal tissues by thiobarbituric acid reaction. Anal Biochem 95(2):351–358. https://pubmed.ncbi.nlm.nih.gov/36810/36810 10.1016/0003-2697(79)90738-3

[24] Wehr NB, Levine RL (2013) Quantification of protein carbonylation. Methods Mol Biol 965:265–281. https://pubmed.ncbi.nlm.nih.gov/23296665/23296665 10.1007/978-1-62703-239-1_18

[25] Soares ER, Barbosa CM, Campagnole-Santos MJ, Santos RAS, Alzamora AC (2017) Hypotensive effect induced by microinjection of Alamandine, a derivative of angiotensin-(1-7), into caudal ventrolateral medulla of 2K1C hypertensive rats. Peptides 96:67–75. https://pubmed.ncbi.nlm.nih.gov/28889964/28889964 10.1016/j.peptides.2017.09.005

[26] de Souza-Neto FP, Carvalho Santuchi M, de Morais E, Silva M, Campagnole-Santos MJ, da Silva RF (2018) Angiotensin-(1-7) and Alamandine on Experimental Models of Hypertension and Atherosclerosis. Curr Hypertens Rep 20(2):17. https://pubmed.ncbi.nlm.nih.gov/29541937/29541937 10.1007/s11906-018-0798-6

[27] Zhao K, Hua D, Yang C, Wu X, Mao Y, Sheng Y, Sun W, Li Y, Kong X, Li P (2023) Nuclear import of Mas-related G protein-coupled receptor member D induces pathological cardiac remodeling. Cell Commun Signal 21(1):181. http://creativecommons.org/licenses/by/4.0/.TheCreativeCommonsPublicDomainDedicationwaiver 37488545 10.1186/s12964-023-01168-3 PMC10364433

[28] Silva MM, de Souza-Neto FP, Jesus ICG, Gonçalves GK, Santuchi MC, Sanches BL, de Alcântara-Leonídio TC, Melo MB, Vieira MAR, Guatimosim S, Santos RAS, da Silva RF (2021) Alamandine improves cardiac remodeling induced by transverse aortic constriction in mice. Am J Physiol Heart Circ Physiol 320(1):H352–H363. https://pubmed.ncbi.nlm.nih.gov/33124885/33124885 10.1152/ajpheart.00328.2020

[29] Wang L, Liu C, Chen X, Li P (2019) Alamandine attenuates long–term hypertension–induced cardiac fibrosis independent of blood pressure. Mol Med Rep 19(6):4553–4560. https://pmc.ncbi.nlm.nih.gov/articles/PMC6522836/31059021 10.3892/mmr.2019.10167 PMC6522836

[30] Jensen MT, Fung K, Aung N, Sanghvi MM, Chadalavada S, Paiva JM, Khanji MY, de Knegt MC, Lukaschuk E, Lee AM, Barutcu A, Maclean E, Carapella V, Cooper J, Young A, Piechnik SK, Neubauer S, Petersen SE (2019) Changes in Cardiac Morphology and Function in Individuals With Diabetes Mellitus: The UK Biobank Cardiovascular Magnetic Resonance Substudy. Circ Cardiovasc Imaging 12(9):e009476. https://www.ahajournals.org/doi/abs/10.1161/CIRCIMAGING.119.009476 31522551 10.1161/CIRCIMAGING.119.009476 PMC7099857

[31] Gella A, Durany N (2009 Jan-Mar) Oxidative stress in Alzheimer disease. Cell Adh Migr 3(1):88–93. https://www.tandfonline.com/action/journalInformation?journalCode=kcam20 10.4161/cam.3.1.7402 PMC2675154 19372765

[32] Lin JH, Walter P, Yen TS (2008) Endoplasmic reticulum stress in disease pathogenesis. Annu Rev Pathol 3:399–425. https://pmc.ncbi.nlm.nih.gov/articles/PMC3653419/18039139 10.1146/annurev.pathmechdis.3.121806.151434 PMC3653419

[33] Clarke BA, Drujan D, Willis MS, Murphy LO, Corpina RA, Burova E, Rakhilin SV, Stitt TN, Patterson C, Latres E, Glass DJ (2007) The E3 Ligase MuRF1 degrades myosin heavy chain protein in dexamethasone-treated skeletal muscle. Cell Metab 6(5):376–385. https://pubmed.ncbi.nlm.nih.gov/17983583/17983583 10.1016/j.cmet.2007.09.009

[34] Tintignac LA, Lagirand J, Batonnet S, Sirri V, Leibovitch MP, Leibovitch SA (2005) Degradation of MyoD mediated by the SCF (MAFbx) ubiquitin ligase. J Biol Chem 280(4):2847–2856. https://pubmed.ncbi.nlm.nih.gov/15531760/15531760 10.1074/jbc.M411346200

[35] Lagirand-Cantaloube J, Offner N, Csibi A, Leibovitch MP, Batonnet-Pichon S, Tintignac LA, Segura CT, Leibovitch SA (2008) The initiation factor eIF3-f is a major target for atrogin1/MAFbx function in skeletal muscle atrophy. EMBO J 27(8):1266-1276 https://www.embopress.org/doi/pdf/10.1038/emboj.2008.52?download=true 10.1038/emboj.2008.52 PMC2367397 18354498

[36] Cabello-Verrugio C, Rivera JC, Garcia D (2017) Skeletal muscle wasting: new role of nonclassical renin-angiotensin system. Curr Opin Clin Nutr Metab Care 20(3):158–163. https://pubmed.ncbi.nlm.nih.gov/28207424/28207424 10.1097/MCO.0000000000000361

[37] Proença AB, Alexandre-Santos B, Giori IG, Alex-Marques JSF, Machado-Santos C, Machado M, Magliano DC, da Nobrega ACL, Frantz EDC (2024) Obesity-induced skeletal muscle remodeling: A comparative analysis of exercise training and ACE-inhibitory drug in male mice. Physiol Rep 12(9):e16025. https://pubmed.ncbi.nlm.nih.gov/38684378/38684378 10.14814/phy2.16025 PMC11058004

