# Supplementary material for: Genetic deletion of MrgD receptor disrupts cardiac protein homeostasis in mice
Source: Mol Biol Rep. 2026 Mar 7;53(1):472. doi: 10.1007/s11033-026-11639-8 (PMC12967435; doi:10.1007/s11033-026-11639-8)
Supplement: Supplementary file 1 — Supplementary Material 1 [file 11033_2026_11639_MOESM1_ESM.docx]

Table S1. Primers used in RT-qPCR assay

| Gene | Primer | |
| --- | --- | --- |
| Col1a1 | Foward | ACGTCCTGGTGAAGTTGGTC |
|  | Reverse | CAGGGAAGCCTCTTTCTCCT |
| Col3a1 | Foward | AGAGAAAGGTGAAGGAGGCC |
|  | Reverse | ACTACCGCGTTCACCCTTTA |
| Nox2 | Foward | CCAATCACTTTGCTGTGCACC |
|  | Reverse | CGCCTATTGTGGTGTTAGGGT |
| Nox4 | Foward | TGCTCATTTGGCTGTCCCTA |
|  | Reverse | ACACAATCCTAGGCCCAACA |
| Grp78 | Foward | TGCGGCCAAGAACCA ACTC |
|  | Reverse | AATGTCTTGGTTTGCCCACCTC |
| Chop | Foward | TATCTCATCCCCAGGAAACG |
|  | Reverse | GGGCACTGACCACTCTGTTT |
| Tbp | Foward | TACAGGTGGCAGCATGAAGTGACA |
|  | Reverse | AACCAACAATCACCAGCAGCAGTG |
